# Ethyl 5-cyano-8-nitro-2,3,4,4a,5,6-hexahydro-1*H*-pyrido[1,2-*a*]quinoline-5-carboxylate

**DOI:** 10.1107/S160053681002283X

**Published:** 2010-06-23

**Authors:** Yapi Marcellin Yapo, Kouakou Michel Konan, Ané Adjou, Adéyolé Timotou, Jules A. Tenon

**Affiliations:** a Laboratoire de Cristallographie et Physique Moléculaire, UFR–SSMT, Université de Cocody, 22 BP 582 Abidjan 22, Côte d’Ivoire; b Laboratoire de Chimie Organique Structurale, UFR–SSMT, Université de Cocody, 22 BP 582 Abidjan 22, Côte d’Ivoire

## Abstract

In the title compound, C_17_H_19_N_3_O_4_, the piperidine ring adopts a chair conformation. The crystal structure features inversion dimers linked by pairs of weak C—H⋯N hydrogen bonds.

## Related literature

For the therapeutic properties of quinoline derivatives, see: Dalla Via *et al.* (2008 ▶); Gasparotto *et al.* (2006 ▶); Ferlin *et al.* (2000 ▶). A similar heterocyclic structure, Mitomycin C, is used in cancer therapy, see: Crooke & Bradner (1976 ▶); Danishefsky & Ciufolini (1984 ▶); Remers (1980 ▶). For related structures, see: Zhuravleva *et al.* (2009 ▶); Oliveira *et al.* (2006 ▶). For ring conformation analysis, see: Cremer & Pople (1975 ▶). For reference bond lengths, see: Allen *et al.* (1987 ▶).
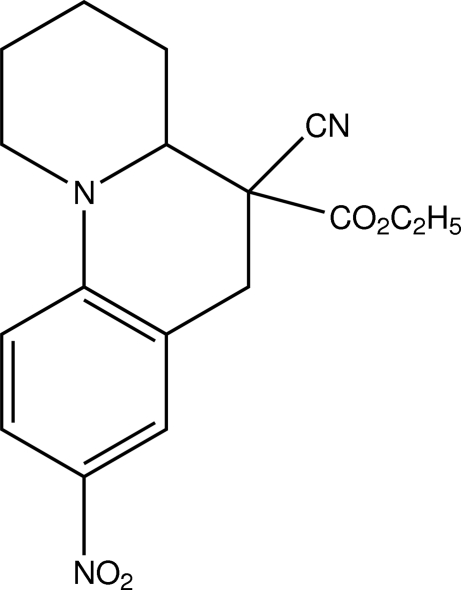


## Experimental

### 

#### Crystal data

C_17_H_19_N_3_O_4_
*M* *_r_* = 329.36
Triclinic, 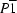

*a* = 8.8257 (4) Å
*b* = 9.2256 (5) Å
*c* = 10.5011 (6) Å
α = 88.246 (4)°
β = 75.089 (2)°
γ = 83.289 (3)°
*V* = 820.57 (8) Å^3^
*Z* = 2
Mo *K*α radiation
μ = 0.10 mm^−1^
*T* = 223 K
0.20 × 0.20 × 0.20 mm

#### Data collection

Nonius KappaCCD diffractometer
10064 measured reflections
4189 independent reflections
2794 reflections with *I* > 2σ(*I*)
*R*_int_ = 0.04

#### Refinement

*R*[*F* ^2^ > 2σ(*F* ^2^)] = 0.055
*wR*(*F* ^2^) = 0.096
*S* = 1.04
2503 reflections
217 parameters
H-atom parameters constrained
Δρ_max_ = 0.21 e Å^−3^
Δρ_min_ = −0.26 e Å^−3^

### 

Data collection: *COLLECT* (Nonius, 2001 ▶); cell refinement: *DENZO*/*SCALEPACK* (Otwinowski & Minor, 1997 ▶); data reduction: *DENZO*/*SCALEPACK*; program(s) used to solve structure: *SIR2004* (Burla *et al.*, 2005 ▶); program(s) used to refine structure: *CRYSTALS* (Betteridge *et al.*, 2003 ▶); molecular graphics: *PLATON* (Spek, 2009 ▶); software used to prepare material for publication: *CRYSTALS*.

## Supplementary Material

Crystal structure: contains datablocks global, I. DOI: 10.1107/S160053681002283X/bq2218sup1.cif

Structure factors: contains datablocks I. DOI: 10.1107/S160053681002283X/bq2218Isup2.hkl

Additional supplementary materials:  crystallographic information; 3D view; checkCIF report

## Figures and Tables

**Table 1:** Hydrogen-bond geometry (Å, °)

*D*—H⋯*A*	*D*—H	H⋯*A*	*D*⋯*A*	*D*—H⋯*A*
C7—H72⋯N3^i^	0.97	2.56	3.492 (3)	161

Symmetry code: (i) 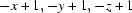.

**Fig. 1.**
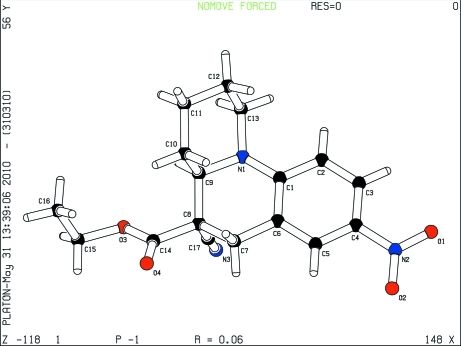
The title compound with displacement ellipsoids drawn at the 50% probability level.

**Fig. 2.**
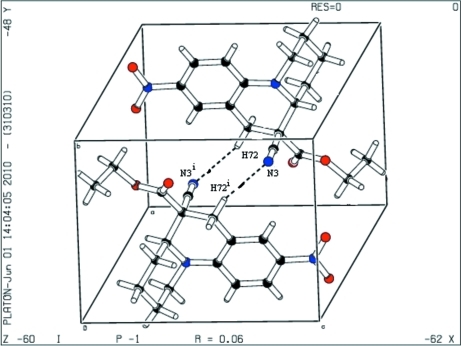
The crystal packing of (I). Hydrogen bonds C-H···N are shown as dashes lines: Symmetry code : (i) -x+1, -y+1, -z+1.

## supplementary crystallographic information

### Comment

Tricyclic quinoline derivatives have diverse and important therapeutic properties (Dalla Via *et al.*, 2008; Gasparotto *et al.*, 2006; Ferlin *et al.*, 2000). These heterocyclic are similar to Mitomycin C which is a powerful antibiotic used in cancerous chemotherapy (Crooke *et al.*, 1976; Remers *et al.*, 1980; Danishefsky *et al.*, 1984). They are also used as intermediate compound to elaborate keratic fiber colorings. Here, we report the single X-ray determination of the title compound C_17_H_19_N_3_O_4_, (I), in order to have a best insight of its structure and then to undertake a study of its possible therapeutic activity. The molecular structure of this compound and its atomic labeling scheme are shown in Figure 1. The bond lenghts distances are within the accepted range (Allen *et al.*, 1987). In (I), there are two coupled rings: quinoline and piperidine rings. The geometrical characteristics relating bond distances in quinoline ring are consistent and present no particularity with the recently reported (Oliveira *et al.*, 2006; Zhuravleva *et al.*, 2009). By least squares planes method, it is observed that carbon atom C8 deviates of -0.4074Å to quinoline cycle plane what proves that quinoline ring is not veritably plane. Concerning piperidine ring, it assumes a chair conformation which the puckering parameters (Cremer & Pople, 1975): θ=7.78°, Q=0.6147Å and Φ=42.46°. The crystal packing is due to the weak hydrogen bonds C-H···N which ensure crystal cohesion (Table 1 and Figure 2).

### Experimental

3.5 g, 10 mmol of malonic arylidene was dissolved in 10 ml of dimethylformamide. The melange was heated to reflux during 24 h. After cooling to ambient temperature, 20 ml of water was added to the melange. After extraction to ethyl acetate (150 ml), the organic layers were dried on magnesium sulfate, filtered and concentrated under reduced pressure. The residue was purified by chromatography on silica gel using hexane/ethyl acetate (80/20) to obtain yellow crystals with 45% yields. The melting point is 424 K

### Refinement

The H atoms were all located in a difference map and then treated as ridings atoms with C—H in the range 0.93–0.98Å and *U*_iso_(H) in the range 1.2–1.5 times *U*_eq_ of the parent atom.

### Crystal data

**Table:**

C_17_H_19_N_3_O_4_	*Z* = 2
*M**_r_* = 329.36	*F*(000) = 348
Triclinic, *P*1	*D*_x_ = 1.333 Mg m^−^^3^
Hall symbol: -P 1	Melting point: 424 K
*a* = 8.8257 (4) Å	Mo *K*α radiation, λ = 0.71073 Å
*b* = 9.2256 (5) Å	Cell parameters from 10064 reflections
*c* = 10.5011 (6) Å	θ = 2–29°
α = 88.246 (4)°	µ = 0.10 mm^−^^1^
β = 75.089 (2)°	*T* = 223 K
γ = 83.289 (3)°	Prism, yellow
*V* = 820.57 (8) Å^3^	0.20 × 0.20 × 0.20 mm

### Data collection

**Table:**

Nonius KappaCCD diffractometer	*R*_int_ = 0.04
graphite	θ_max_ = 29.1°, θ_min_ = 2.0°
φ and ω scans	*h* = 0→12
10064 measured reflections	*k* = −11→12
4189 independent reflections	*l* = −13→14
2794 reflections with *I* > 2σ(*I*)	

### Refinement

**Table:**

Refinement on *F*^2^	Primary atom site location: structure-invariant direct methods
Least-squares matrix: full	Hydrogen site location: inferred from neighbouring sites
*R*[*F*^2^ > 2σ(*F*^2^)] = 0.055	H-atom parameters constrained
*wR*(*F*^2^) = 0.096	*w* = 1/[σ^2^(*F*^2^) + (0.02*P*)^2^ + 0.5*P*], where *P* = (max(*F*_o_^2^,0) + 2*F*_c_^2^)/3
*S* = 1.04	(Δ/σ)_max_ = 0.0002
2503 reflections	Δρ_max_ = 0.21 e Å^−^^3^
217 parameters	Δρ_min_ = −0.26 e Å^−^^3^
0 restraints	

### Fractional atomic coordinates and isotropic or equivalent isotropic displacement parameters (Å^2^)

**Table:**

	*x*	*y*	*z*	*U*_iso_*/*U*_eq_	
C1	0.70920 (19)	0.86006 (18)	0.50748 (17)	0.0241	
N1	0.70324 (17)	0.85963 (14)	0.63954 (14)	0.0268	
O3	0.66155 (17)	0.46173 (14)	0.84947 (13)	0.0393	
C9	0.7451 (2)	0.72488 (18)	0.70764 (17)	0.0256	
C2	0.6873 (2)	0.99129 (19)	0.43830 (18)	0.0287	
C6	0.7271 (2)	0.72669 (18)	0.43802 (17)	0.0269	
C5	0.7160 (2)	0.72795 (19)	0.30929 (18)	0.0308	
C4	0.6920 (2)	0.8595 (2)	0.24574 (17)	0.0311	
O4	0.8187 (2)	0.35165 (15)	0.66714 (15)	0.0548	
O2	0.6821 (2)	0.74186 (19)	0.05771 (16)	0.0639	
C3	0.6790 (2)	0.99068 (19)	0.30939 (18)	0.0308	
C7	0.7604 (2)	0.58338 (19)	0.50323 (18)	0.0325	
C8	0.6879 (2)	0.59435 (18)	0.65159 (17)	0.0278	
C10	0.9210 (2)	0.7072 (2)	0.70064 (19)	0.0327	
N3	0.3802 (2)	0.63652 (18)	0.69548 (19)	0.0450	
N2	0.6797 (2)	0.8591 (2)	0.11110 (17)	0.0436	
O1	0.6673 (2)	0.97711 (19)	0.05381 (15)	0.0652	
C13	0.7303 (2)	0.98771 (19)	0.70769 (18)	0.0310	
C17	0.5143 (2)	0.61781 (18)	0.67811 (19)	0.0322	
C12	0.9039 (2)	0.9820 (2)	0.70523 (19)	0.0346	
C14	0.7321 (2)	0.45278 (19)	0.72194 (19)	0.0328	
C11	0.9606 (2)	0.8393 (2)	0.7643 (2)	0.0358	
C15	0.6946 (3)	0.3372 (2)	0.9327 (2)	0.0461	
C16	0.8465 (4)	0.3441 (3)	0.9667 (3)	0.0711	
H91	0.6874	0.7364	0.8009	0.0307*	
H51	0.7270	0.6393	0.2640	0.0374*	
H31	0.6639	1.0787	0.2640	0.0358*	
H71	0.8751	0.5568	0.4875	0.0395*	
H72	0.7193	0.5065	0.4655	0.0388*	
H101	0.9454	0.6173	0.7460	0.0386*	
H102	0.9806	0.7000	0.6083	0.0399*	
H122	0.9191	1.0653	0.7543	0.0424*	
H121	0.9656	0.9890	0.6138	0.0425*	
H112	1.0737	0.8312	0.7531	0.0436*	
H111	0.9082	0.8405	0.8586	0.0444*	
H152	0.6075	0.3476	1.0124	0.0547*	
H151	0.6960	0.2467	0.8845	0.0545*	
H162	0.8619	0.2658	1.0269	0.0858*	
H161	0.8453	0.4366	1.0086	0.0858*	
H163	0.9346	0.3328	0.8875	0.0858*	
H21	0.6783	1.0810	0.4814	0.0340*	
H131	0.6679	0.9883	0.7985	0.0380*	
H132	0.6990	1.0760	0.6639	0.0380*	

### Atomic displacement parameters (Å^2^)

**Table:**

	*U*^11^	*U*^22^	*U*^33^	*U*^12^	*U*^13^	*U*^23^
C1	0.0202 (8)	0.0264 (9)	0.0259 (9)	−0.0011 (6)	−0.0068 (7)	−0.0010 (7)
N1	0.0338 (9)	0.0213 (7)	0.0269 (8)	0.0003 (6)	−0.0122 (7)	−0.0028 (6)
O3	0.0480 (9)	0.0337 (7)	0.0313 (8)	0.0038 (6)	−0.0057 (6)	0.0066 (6)
C9	0.0293 (9)	0.0242 (8)	0.0237 (9)	−0.0009 (7)	−0.0087 (7)	0.0007 (7)
C2	0.0264 (9)	0.0269 (9)	0.0316 (10)	−0.0020 (7)	−0.0061 (8)	0.0012 (7)
C6	0.0268 (9)	0.0274 (9)	0.0258 (9)	−0.0008 (7)	−0.0066 (7)	0.0000 (7)
C5	0.0332 (10)	0.0331 (10)	0.0262 (9)	−0.0035 (8)	−0.0076 (8)	−0.0024 (7)
C4	0.0301 (10)	0.0422 (11)	0.0206 (9)	−0.0055 (8)	−0.0058 (7)	0.0036 (7)
O4	0.0801 (12)	0.0323 (8)	0.0403 (9)	0.0185 (8)	−0.0056 (8)	0.0011 (6)
O2	0.0985 (15)	0.0666 (11)	0.0350 (9)	−0.0240 (10)	−0.0255 (9)	−0.0007 (8)
C3	0.0245 (9)	0.0345 (10)	0.0313 (10)	−0.0025 (7)	−0.0048 (8)	0.0086 (8)
C7	0.0452 (11)	0.0265 (9)	0.0259 (10)	0.0021 (8)	−0.0115 (9)	−0.0037 (7)
C8	0.0326 (10)	0.0233 (9)	0.0276 (10)	0.0006 (7)	−0.0097 (8)	0.0001 (7)
C10	0.0302 (10)	0.0314 (10)	0.0361 (11)	0.0007 (8)	−0.0101 (8)	0.0039 (8)
N3	0.0402 (11)	0.0384 (10)	0.0596 (12)	−0.0061 (8)	−0.0174 (9)	−0.0034 (8)
N2	0.0455 (11)	0.0574 (12)	0.0267 (9)	−0.0062 (9)	−0.0076 (8)	0.0059 (8)
O1	0.0931 (14)	0.0654 (11)	0.0342 (9)	0.0056 (10)	−0.0195 (9)	0.0154 (8)
C13	0.0372 (11)	0.0257 (9)	0.0320 (10)	−0.0002 (8)	−0.0132 (8)	−0.0046 (7)
C17	0.0422 (12)	0.0223 (9)	0.0349 (10)	−0.0051 (8)	−0.0141 (9)	−0.0009 (7)
C12	0.0362 (11)	0.0338 (10)	0.0358 (11)	−0.0078 (8)	−0.0110 (9)	−0.0017 (8)
C14	0.0400 (11)	0.0265 (9)	0.0321 (10)	−0.0015 (8)	−0.0105 (9)	0.0009 (8)
C11	0.0282 (10)	0.0431 (11)	0.0378 (11)	−0.0062 (8)	−0.0108 (8)	0.0019 (9)
C15	0.0573 (14)	0.0401 (12)	0.0364 (12)	0.0018 (10)	−0.0088 (10)	0.0141 (9)
C16	0.0735 (19)	0.086 (2)	0.0595 (17)	−0.0018 (15)	−0.0330 (15)	0.0224 (14)

### Geometric parameters (Å, °)

**Table:**

C1—N1	1.374 (2)	C7—H72	0.971
C1—C2	1.412 (2)	C8—C17	1.476 (3)
C1—C6	1.422 (2)	C8—C14	1.541 (2)
N1—C9	1.472 (2)	C10—C11	1.525 (3)
N1—C13	1.474 (2)	C10—H101	0.973
O3—C14	1.324 (2)	C10—H102	0.977
O3—C15	1.469 (2)	N3—C17	1.143 (2)
C9—C8	1.546 (2)	N2—O1	1.235 (2)
C9—C10	1.524 (3)	C13—C12	1.520 (3)
C9—H91	0.983	C13—H131	0.970
C2—C3	1.375 (3)	C13—H132	0.970
C2—H21	0.941	C12—C11	1.524 (3)
C6—C5	1.380 (2)	C12—H122	0.979
C6—C7	1.504 (2)	C12—H121	0.979
C5—C4	1.388 (3)	C11—H112	0.969
C5—H51	0.941	C11—H111	0.979
C4—C3	1.379 (3)	C15—C16	1.482 (4)
C4—N2	1.446 (2)	C15—H152	0.979
O4—C14	1.194 (2)	C15—H151	0.987
O2—N2	1.229 (2)	C16—H162	0.966
C3—H31	0.942	C16—H161	0.969
C7—C8	1.526 (2)	C16—H163	0.980
C7—H71	0.985		
			
N1—C1—C2	121.61 (15)	C11—C10—H101	111.3
N1—C1—C6	120.57 (15)	C9—C10—H102	109.0
C2—C1—C6	117.69 (16)	C11—C10—H102	109.9
C1—N1—C9	121.40 (13)	H101—C10—H102	109.1
C1—N1—C13	122.69 (14)	C4—N2—O2	119.05 (17)
C9—N1—C13	109.99 (13)	C4—N2—O1	118.60 (18)
C14—O3—C15	117.40 (15)	O2—N2—O1	122.36 (18)
N1—C9—C8	109.45 (14)	N1—C13—C12	110.21 (14)
N1—C9—C10	110.06 (14)	N1—C13—H131	109.3
C8—C9—C10	114.36 (14)	C12—C13—H131	109.3
N1—C9—H91	107.0	N1—C13—H132	109.3
C8—C9—H91	108.0	C12—C13—H132	109.3
C10—C9—H91	107.7	H131—C13—H132	109.4
C1—C2—C3	121.40 (16)	C8—C17—N3	178.4 (2)
C1—C2—H21	119.3	C13—C12—C11	110.80 (15)
C3—C2—H21	119.3	C13—C12—H122	109.2
C1—C6—C5	120.13 (16)	C11—C12—H122	110.4
C1—C6—C7	120.45 (15)	C13—C12—H121	109.0
C5—C6—C7	119.41 (15)	C11—C12—H121	109.0
C6—C5—C4	120.19 (16)	H122—C12—H121	108.5
C6—C5—H51	119.8	C8—C14—O3	110.12 (15)
C4—C5—H51	120.0	C8—C14—O4	123.73 (18)
C5—C4—C3	120.93 (17)	O3—C14—O4	126.15 (17)
C5—C4—N2	119.57 (17)	C10—C11—C12	111.70 (16)
C3—C4—N2	119.49 (17)	C10—C11—H112	108.7
C4—C3—C2	119.60 (16)	C12—C11—H112	110.4
C4—C3—H31	119.5	C10—C11—H111	109.4
C2—C3—H31	120.9	C12—C11—H111	107.7
C6—C7—C8	110.18 (14)	H112—C11—H111	108.9
C6—C7—H71	110.2	O3—C15—C16	110.69 (19)
C8—C7—H71	108.6	O3—C15—H152	104.8
C6—C7—H72	110.0	C16—C15—H152	110.0
C8—C7—H72	110.7	O3—C15—H151	108.1
H71—C7—H72	107.2	C16—C15—H151	111.3
C9—C8—C7	109.74 (14)	H152—C15—H151	111.7
C9—C8—C17	108.75 (14)	C15—C16—H162	109.3
C7—C8—C17	109.52 (15)	C15—C16—H161	110.0
C9—C8—C14	109.75 (14)	H162—C16—H161	109.0
C7—C8—C14	111.10 (14)	C15—C16—H163	110.6
C17—C8—C14	107.93 (15)	H162—C16—H163	108.3
C9—C10—C11	109.16 (14)	H161—C16—H163	109.6
C9—C10—H101	108.3		

### Hydrogen-bond geometry (Å, °)

**Table:**

*D*—H···*A*	*D*—H	H···*A*	*D*···*A*	*D*—H···*A*
C7—H72···N3^i^	0.97	2.56	3.492 (3)	161

Symmetry codes: (i) −*x*+1, −*y*+1, −*z*+1.
